# Algorithm development and the clinical and economic burden of Cushing’s disease in a large US health plan database

**DOI:** 10.1007/s11102-015-0695-9

**Published:** 2015-12-14

**Authors:** Tanya Burton, Elisabeth Le Nestour, Maureen Neary, William H. Ludlam

**Affiliations:** Optum, 950 Winter Street, Waltham, MA 02451 USA; InVentiv Health Clinical, 41 rue des 3 Fontanot, 92000 Nanterre, France; Novartis Pharmaceuticals Corporation, One Health Plaza, East Hanover, NJ 07936 USA

**Keywords:** Cushing’s disease, Case-finding algorithm, Comorbidities, Health care costs

## Abstract

**Purpose:**

This study aimed to develop an algorithm to identify patients with CD, and quantify the clinical and economic burden that patients with CD face compared to CD-free controls.

**Methods:**

A retrospective cohort study of CD patients was conducted in a large US commercial health plan database between 1/1/2007 and 12/31/2011. A control group with no evidence of CD during the same time was matched 1:3 based on demographics. Comorbidity rates were compared using Poisson and health care costs were compared using robust variance estimation.

**Results:**

A case-finding algorithm identified 877 CD patients, who were matched to 2631 CD-free controls. The age and sex distribution of the selected population matched the known epidemiology of CD. CD patients were found to have comorbidity rates that were two to five times higher and health care costs that were four to seven times higher than CD-free controls.

**Conclusion:**

An algorithm based on eight pituitary conditions and procedures appeared to identify CD patients in a claims database without a unique diagnosis code. Young CD patients had high rates of comorbidities that are more commonly observed in an older population (e.g., diabetes, hypertension, and cardiovascular disease). Observed health care costs were also high for CD patients compared to CD-free controls, but may have been even higher if the sample had included healthier controls with no health care use as well. Earlier diagnosis, improved surgery success rates, and better treatments may all help to reduce the chronic comorbidity and high health care costs associated with CD.

**Electronic supplementary material:**

The online version of this article (doi:10.1007/s11102-015-0695-9) contains supplementary material, which is available to authorized users.

## Introduction

Cushing’s disease (CD) is a rare and debilitating condition that is caused by a pituitary adenoma secreting excessive adrenocorticotropic hormone (ACTH). It occurs mainly in young women of child-bearing age [1] and is one of several causes of hypercortisolism (also known as Cushing’s syndrome). The signs and symptoms of hypercortisolism include obesity, fatigue, depression, muscle weakness, hypertension, headache, diabetes, easy bruising, stria, and osteoporotic fractures. Many of these conditions are common in the general population, which often obscures CD and delays its diagnosis [2]. Subsequently, with continued exposure to high endogenous cortisol levels, health-related quality of life (HRQoL) is impaired and the risk of pre-mature death increases [2].

Although surgery and optimal follow-up may normalize mortality, remission rates at best may range from 65 to 90 % and relapse is common [3]. Previously, effective treatments were off-label, but recently some new medications have been approved and some off-label medications are now being studied for approval [4]. If not treated adequately, the risk of morbidity and mortality associated with CD may persist and remain high. Consequently, the economic burden associated with CD may also be significant resulting from its chronic nature and impact on major metabolic systems (e.g., glucose and lipid regulation, cardiovascular, immunity, skin, and skeletal). To date, there is limited literature in the US comparing the comorbidities and health care costs of CD patients and CD-free controls.

Analyzing administrative claims from a health care database is a cost-effective way to study a rare disease such as CD. Collected for billing purposes, health care claims include codes for medical conditions and prescribed treatments and procedures, which make them a valuable resource for studying real-world treatment patterns, health care outcomes and costs [5]. However, the *International Classification of Diseases, 9th Revision, Clinical Modification* (ICD-9-CM) system does not include a unique diagnosis code for CD, which complicates the process of identifying CD patients in claims. To contribute to the literature, the objectives of this research were to (1) develop an algorithm to identify patients with CD in an administrative claims database without a unique diagnosis code, and (2) quantify the clinical and economic burden that patients with CD face compared to CD-free controls in a large US managed care database.

## Methods

### Study design and sample

This was a retrospective cohort study using administrative claims from a large health insurance database between July 1, 2006 and June 30, 2012 (study period). The database includes over 12.9 million commercial health plan enrollees annually with medical and pharmacy benefits. Health plans included in the database are geographically diverse across the US.

Medical (professional and facility) claims in the database include ICD-9-CM diagnosis and procedure codes, *Current Procedural Terminology, Version 4* (CPT-4) procedure codes, *Healthcare Common Procedure Coding System* (HCPCS) procedure codes, site of service codes, and health plan- and patient-paid amounts. Outpatient pharmacy claims include National Drug Codes (NDC) for dispensed medications, dosage form, fill date, and health plan- and patient-paid amounts. All administrative claims data are de-identified and compliant with the provisions of the Health Insurance Portability and Accountability Act of 1996.

### Cushing’s disease case identification

An algorithm was developed using medical expert opinion to identify patients with CD in administrative claims. Starting with an ICD-9-CM diagnosis code for Cushing’s syndrome (CS), the broader spectrum of etiologies encompassing CD, two endocrinologists evaluated 18 pituitary conditions and procedures to distinguish CD from CS. The result was to identify children (<18 years) and adult commercial health plan enrollees as having CD if they had (1) at least one medical claim with a CS diagnosis code (ICD-9-CM: 255.0) and (2) at least one of eight selected pituitary conditions and procedures (Table 1). CS and pituitary diagnosis codes on medical claims from laboratories and diagnostic testing centers were not examined for selection, as they may have included “rule-out” procedures for a diagnosis not yet confirmed. The identification period was from January 1, 2007 through December 31, 2011 and the index date was defined as the first date for CS, a CD-related condition (pituitary tumor or disorder), or CD-related procedure (hypophysectomy, radiation, or bilateral inferior petrosal sinus sampling) during the identification period. The CD-related condition or procedure on the index date was defined as the index CD event. Selected CD patients were required to be continuously enrolled with medical and pharmacy benefits for 6 months before (pre-index period) and at least 6 months after the index date (post-index period) until the earliest of death (as evidenced by hospital discharge claims or Social Security Administration death records), disenrollment from the health plan, or the study cut-off date (June 30, 2012).

**Table 1:** Cushing disease-related conditions and procedures

Pituitary conditions	Pituitary procedures
Pituitary neoplasm	Hypophysectomy
Pituitary disorders:	Cranial stereotactic radiosurgery
Hyperfunction	Bilateral inferior petrosal sinus catheterization with cortisol or adrenocorticotropic hormone sampling
Other anterior pituitary disorder	
Hypothalamic control of anterior pituitary	
Syndromes of diencephalohypophyseal origin	

Diagnosis and procedure codes are shown in Supplement Table A

### Disease-free control identification

Children and adult health plan enrollees without claims for CS and CD-related conditions and procedures during the study period were included in this study as CD-free controls. Controls were required to have at least one medical claim for any other condition during the identification period, and were observed during a similar time period starting in the same index year as the identified CD patients. The index date for controls was defined as the first claim date for a medical service during the identification period. Each CD patient was matched to three randomly selected CD-free controls by age, sex, geographic region, and index year.

## Measurements

### Demographic and pre-index clinical characteristics

Demographic variables included age, sex, and the US census region of the health plan. General comorbid conditions were identified using the Clinical Classifications Software managed by the Agency for Healthcare Research and Quality (AHRQ) [6]. This software generates indicator variables for specific disease conditions based on the presence of ICD-9-CM diagnosis codes in the database. The top five comorbid conditions during the pre-index period were identified for children and adults.

### Clinical burden

Incident comorbidities and complications for CD patients and CD-free controls were identified during the post-index period. Incidence was defined as the first occurrence of a medical claim for a comorbidity or complication during the post-index period, given that no such medical claims were present during the 6-month pre-index period. The selected medical conditions were derived from a list presented in Feelders [7] and Clayton [8].

### Economic burden

Economic outcomes included all-cause health care resource utilization and costs during the post-index period. Costs were computed as the sum of health plan- and patient-paid amounts for all inpatient and outpatient medical services delivered during the post-index period, and were adjusted for inflation to 2011 dollars using the annual medical care component of the Consumer Price Index [9]. Per patient per month (PPPM) counts and costs of all-cause health care resource utilization were computed to account for variable follow-up.

### Statistical analysis

All study variables were summarized descriptively. Categorical variables were summarized with frequencies and percentages and continuous variables with means, standard deviations (SD), and medians. Rao-Scott Chi square and robust variance estimation were performed to assess group differences in categorical and continuous measures, respectively. Clustered p-values were calculated to control for within-match correlation.

Incidence rates for the comorbidities and complications were calculated by dividing the number of “new” cases (i.e., where no such medical claims existed during the pre-index period) by 100 person-years at risk. The time at risk was calculated as the number of days from the index date until the date of the first claim for the medical condition. Disease rates between CD patients and CD-free controls were compared using incidence rate ratios (IRR) and 95 % confidence intervals (CI) from the Poisson distribution with a logarithmic link function and logarithmic person-time offset to account for the variable observation time.

All analyses were performed using SAS v9.2 (SAS Institute, Cary, NC). A *p* value less than 0.05 and a 95 % confidence interval not containing 1 were used to signify statistical significance.

## Results

**Fig. 1:**
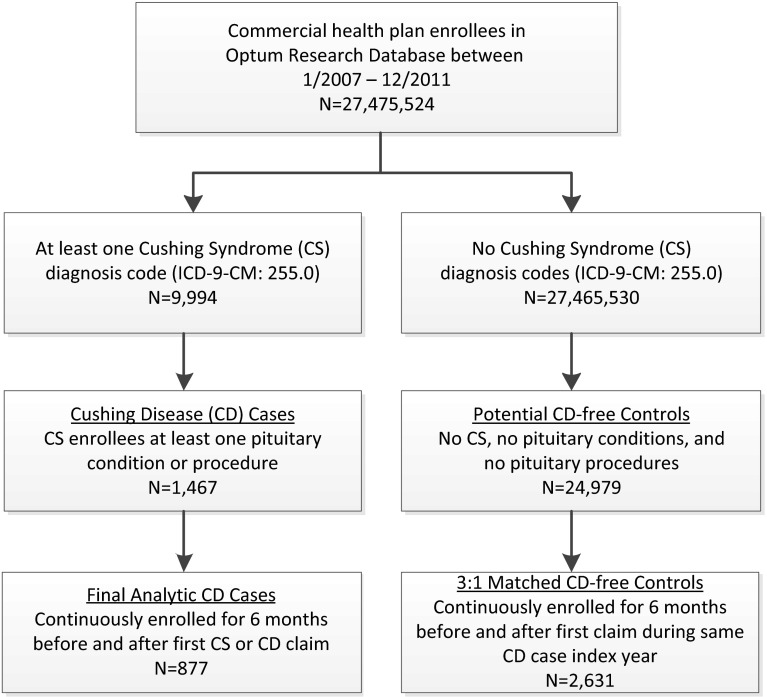
Sample identification

A flow chart of the sample selection is presented in Fig. 1. Of the approximately 27 million commercial health plan enrollees in the database from January 1, 2007 through December 31, 2011, 9994 had at least one medical claim with a CS diagnosis code; of which 1467 (15 %) had a pituitary condition or procedure suggestive of CD. After applying the remaining selection criteria, a total of 877 CD patients were matched to 2631 CD-free controls.

### Demographic and pre-index clinical characteristics

Table 2 presents the demographic and pre-index clinical characteristics. The mean age was 42 years as of the index CD event, and 75 % were women. The geographic distribution matched the health plan with 16 % in the Midwest, 37 % in the Northeast, 36 % in the South, and 10 % in the West.

**Table 2:** Demographics and pre-index clinical characteristics

	Total (N = 3508)	CD (N = 877)	CD-free (N = 2631)	*p* value
Sex, n (%)				
Female	2640 (75.26)	660 (75.26)	1980 (75.26)	–
Male	868 (24.74)	217 (24.74)	651 (24.74)	
Age, n (%)				
Children (1–17 years)	172 (4.90)	43 (4.90)	129 (4.90)	–
Adults (18+ years)	3336 (95.10)	834 (95.10)	2502 (95.10)	
Age, years, mean ± SD	42 ± 14	42 ± 14	42 ± 14	–
Geographic location, n (%)				
Midwest	564 (16.08)	141 (16.08)	423 (16.08)	–
Northeast	1308 (37.29)	327 (37.29)	981 (37.29)	–
South	1268 (36.15)	317 (36.15)	951 (36.15)	–
West	368 (10.49)	92 (10.49)	276 (10.49)	–
Top 5 pre-index AHRQ comorbidities for children, n (%)				
Respiratory infections	58 (33.72)	20 (46.51)	38 (29.46)	0.051
Metabolic disorders	35 (20.35)	27 (62.79)	8 (6.20)	<0.001
Neurological disorders (non-traumatic)	32 (18.60)	25 (58.14)	7 (5.43)	<0.001
Skin conditions	28 (16.28)	18 (41.86)	10 (7.75)	<0.001
Psychiatric/mental disorders	27 (15.70)	11 (25.58)	16 (12.40)	0.087
Top 5 pre-index AHRQ comorbidities for adults, n (%)				
Urogenital disorders	1110 (33.27)	407 (48.80)	703 (28.10)	<0.001
Musculoskeletal disorders (non-traumatic)	818 (24.52)	309 (37.05)	509 (20.34)	<0.001
Endocrine disorders (non-diabetes)	693 (20.77)	492 (58.99)	201 (8.03)	<0.001
Respiratory infections	664 (19.90)	225 (26.98)	439 (17.55)	<0.001
Hypertension	662 (19.84)	278 (33.33)	384 (15.35)	<0.001
Post-index observation period, years, mean ± SD [min, median, max]	2.1 ± 1.4 [0.5, 1.6, 5.5]	2.6 ± 1.5 [0.5, 2.4, 5.5]	1.9 ± 1.3 [0.5, 1.5, 5.5]	<0.001

*SD* standard deviation

Among the top five AHRQ comorbidities identified during the pre-index period, prevalence was 1.5–10 times higher among CD patients than CD-free controls. The top 5 conditions differed between children and adults. The top conditions among children with CD included: metabolic disorders, non-traumatic neurological disorders, skin conditions and psychiatric/mental disorders. The top conditions among adults with CD included: urogenital disorders, non-traumatic musculoskeletal disorders, endocrine disorders (non-diabetes), and hypertension. Both children and adults with CD had high proportions of respiratory infections (47 and 27 %, respectively) during the pre-index period. Total observation time following the index CD event ranged from 6 months to 5.5 years, with a median of 2.4 years for CD patients and 1.5 years for CD-free controls.

### Incremental clinical burden

Incidence rate ratios in Table 3 show that rates of new comorbidity claims remained high for CD patients following the index CD event. Compared to CD-free controls, CD patients had five times the rate of osteoporosis and diabetes, four times the rate of cardiovascular disease, liver disease, and obesity, three times the rate of hypertension, depression, and mood disorders, and twice the rate of dyslipidemia, menstrual abnormalities, and acne. Among children, few comorbidities and complications were observed. At most, 13 children with CD and 5 CD-free controls had claims for abnormal weight gain during the post-index period (IRR 9.1, 95 % CI 2.9–28.4).

**Table 3:** Post-index CD-related comorbid conditions (incidence rates per 100 person-years)

	CD (N = 877)			CD-free (N = 2631)			With clustering		
	Events	Person-years	Rate per 100	Events	Person-years	Rate per 100	Ratio per 100	Lower 95 % CI	Upper 95 % CI
Musculoskeletal									
Vertebral fracture	14	2266	0.6177	9	4887	0.1841	3.6967	1.6069	8.5041
Pathologic fracture	10	2289	0.4368	1	4903	0.0204	19.2745	2.4300	152.8805
Muscle weakness	65	2163	3.0046	22	4861	0.4526	7.5682	4.5271	12.6522
Osteoporosis	138	1877	7.3520	67	4726	1.4176	4.9953	3.6709	6.7976
Cardiovascular									
Cerebrovascular accident	32	2228	1.4363	9	4885	0.1842	7.7964	3.7640	16.1489
Cardiovascular disease	207	1569	13.1931	169	4404	3.8372	3.6866	2.9863	4.5511
Hypertension	223	1087	20.5111	295	3724	7.9207	2.9111	2.3901	3.5456
Acute myocardial infarction	12	2281	0.5260	6	4891	0.1227	4.2876	1.6038	11.4623
Endocrine/metabolic									
Diabetes (incl. impaired glucose tolerance)	172	1578	10.9009	103	4481	2.2984	5.2234	4.0193	6.7881
Dyslipidemia	232	1110	20.8984	405	3482	11.6299	2.0073	1.6807	2.3973
Menstrual abnormality^a^	180	1088	16.5452	263	3038	8.6565	1.9608	1.6134	2.3829
Impaired libido/impotence	22	2218	0.9919	16	4875	0.3282	3.0768	1.6744	5.6540
Obesity	176	1701	10.3459	128	4561	2.8062	3.7265	2.9284	4.7423
Liver disease (acute or chronic)	141	1873	7.5295	94	4684	2.0069	4.0495	3.0834	5.3182
Elevated liver enzymes	34	2202	1.5443	30	4855	0.6180	2.9989	1.7910	5.0212
Malnutrition	20	2250	0.8889	6	4892	0.1226	7.2474	2.9002	18.1103
Mental health									
Depression	171	1599	10.6916	185	4256	4.3467	2.6286	2.1130	3.2700
Cognitive impairment	20	2264	0.8836	4	4897	0.0817	13.7019	4.0339	46.5416
Anxiety	180	1714	10.5023	220	4236	5.1932	2.2551	1.8258	2.7855
Mood disorders	99	1906	5.1947	83	4633	1.7914	2.9922	2.2088	4.0533
Other									
Acne	67	2088	3.2091	88	4697	1.8735	1.9325	1.3850	2.6964
Hirsutism	43	2123	2.0259	8	4892	0.1635	10.0839	4.7417	21.4449
Non-healing surgical would (proxy - poor skin healing)	7	2290	0.3057	2	4903	0.0408	7.4928	1.8713	30.0014
Infections	143	1848	7.7382	173	4536	3.8141	2.1748	1.7170	2.7547
Pediatric conditions^b^									
Short stature	1	29,477	0.0034	0	91,964	0.0000	–	–	–
Delayed sexual development/puberty	3	36,392	0.0082	1	91,881	0.0011	5.0495	0.4568	55.8186
Precocious sexual development/puberty	3	38,664	0.0078	0	92,165	0.0000	–	–	–
Abnormal weight gain	13	23,469	0.0554	5	89,296	0.0056	9.1316	2.9363	28.3983
Overweight	2	39,712	0.0050	1	91,123	0.0011	4.5892	0.4187	50.2984
Failure to thrive	1	39,029	0.0026	0	92,165	0.0000	–	–	–

^a^Females only, ^b^ Children only

### Incremental economic burden

Table 4 presents the mean PPPM counts and costs of health care resource utilization during the post-index period. The mean number of PPPM health care visits was two to four times higher for CD patients than CD-free controls (ambulatory visits: 2.55 vs. 0.86, *p* < 0.001; emergency department visits: 0.12 vs. 0.04, *p* < 0.001; and inpatient admissions: 0.04 vs. 0.01, *p* < 0.001). Total mean PPPM all-cause health care costs were also higher for CD patients than CD-free controls, with an average cost difference of nearly $3000 ($3232 vs. $489, *p* < 0.001). Total costs were driven primarily by medical costs, which accounted for 87 and 79 % of total costs for CD patients and CD-free controls, respectively. On average, medical costs were nearly seven times higher for CD patients than CD-free controls ($2800 vs. $384, *p* < 0.001), and average PPPM pharmacy costs were four times higher for CD patients than CD-free controls ($432 vs. $104, *p* < 0.001).

**Table 4:** Post-index per patient per month (PPPM) count and costs of all-cause health care utilization

	CD (N = 877)	CD-free (N = 2631)	*p* value
PPPM Count, mean (SD)			
Ambulatory	2.55 (2.04)	0.86 (1.00)	<0.001
Outpatient hospital	0.87 (1.03)	0.22 (0.39)	<0.001
Physician office	1.76 (1.52)	0.66 (0.82)	<0.001
Emergency department	0.12 (0.26)	0.04 (0.19)	<0.001
Inpatient hospital	0.04 (0.09)	0.01 (0.03)	<0.001
PPPM Costs, US$, mean (SD) [median]			
Total all-cause health care costs	3232 (6958) [1433]	489 (1247) [184]	<0.001
Medical costs	2800 (6788) [1067]	384 (1079) [121]	<0.001
Pharmacy costs	432 (722) [162]	104 (515) [22]	<0.001

## Discussion

This retrospective analysis of 877 CD patients and 2631 matched CD-free controls was performed using administrative claims data from a large health plan database in the United States. The study developed an algorithm to identify CD patients in the absence of a unique ICD-9 diagnosis code and evaluated the incremental clinical and economic burden of CD compared to CD-free controls. The age and sex distribution of the selected sample matched the known epidemiology of CD [4], suggesting that CD patients may be identifiable in administrative claims using an algorithm based on CD-related diagnosis and procedure codes. In addition, CD patients were found to have a high proportion of comorbidity during the 6-month pre-index period, suggesting that a number of CD patients were likely diagnosed with CD prior to study entry. Claims data, collected for billing purposes, often do not include key measures for clinical research such as the date of diagnosis or patients’ health status. As a result, the pituitary conditions and procedures used in this study to identify CD patients may continue to be relevant and important to consider even after the adoption of the new ICD-10 coding system, which will include for the first time a unique diagnosis code for CD as well as other rare conditions.

During the post-index period, CD patients were found to have higher rates of comorbidities and higher health care resource utilization and costs than CD-free controls. In fact, these young CD patients had comorbidities (e.g., diabetes, hypertension, and cardiovascular disease) and associated costs that are more commonly observed in an older population. Published data on the health and costs of treating CD patients are limited in the US. Although our research methods differed slightly, the findings reported here are consistent with prior studies that in general show CD is a debilitating condition associated with high comorbidity and health care costs [10, 11].

The occurrence of multiple comorbidities along with cortisol-induced anxiety and mood disorders can be distressing for anyone but especially so for patients with CD in their prime reproductive and earning years. Appropriate use of medical therapies to control hypercortisolism has been shown to improve the quality of life for CD patients [12]. However, with variable surgical remission rates between 65 and 90 % [3], increasing the rate of cure with the first surgery may have the highest impact for decreasing future morbidity and costs.

### Limitations

There are several limitations to keep in mind when interpreting the results of this study. First, as with all studies analyzing retrospective billing data, there are limits to how well these data can accurately capture an individual’s medical history. While the conditions and procedures used to create the CD algorithm may meet face validity, medical charts should also be examined to confirm the final CD assignments. Without this confirmation, it is possible that patients with ectopic CS and a petrosal sinus sampling (IPSS), for example, may have been incorrectly included in the CD sample.

Second, it is possible that this study underestimated the clinical and economic burden of CD. The actual incidence of comorbidity may be higher than observed as the date of CD diagnosis is not available in administrative claims. CD is difficult to diagnose and diagnosis can take on average 2–5 years [7]. As a result, additional comorbidities may have been present prior to study entry but not yet attributed to hypercortisolism. For example, diabetes at first may be associated with being overweight, but later, as the intensity of the hypercortisolism increases, realized to be a sign of CD, which means claims during the pre-index period or earlier may not have been properly identified as relating to CD. Consequently, our cost estimates may be conservative as well, as costs between the diagnosis and study entry dates are not included and costs prior to the first claim for CD may have been misattributed to other causes.

Third, the study included controls with at least one medical visit during the identification period. Thus, our control population may reflect a higher rate of comorbidity and health care costs than if a healthier cohort with no health care use was included in the study as well, which suggests another reason the actual clinical and economic burden of CD may be larger than observed.

Fourth, CD patients are typically women of childbearing age and the societal costs of pregnancy delays and risks associated with the comorbidities and complications of CD are not included in this analysis. Instead, only the direct medical costs of the health plans and patients enrolled with one health insurer in the US are presented.

Fifth, the study data came from a commercially insured managed care population and the results are primarily applicable to patient populations who receive their care through similar health delivery systems. The health plans included in the database cover a wide geographic distribution across the US, which suggest the results may be generalizable to comparable managed care populations on a national level.

Notwithstanding these limitations, the use of an administrative claims database provided access to a large and diverse patient population from which an adequate sample of patients with CD, a rare disease, could be identified and evaluated for real-world treatment patterns on a national scale.

## Conclusion

A case-finding algorithm based on eight pituitary conditions and procedures appeared to identify CD patients in an administrative claims database without a unique diagnosis code, and the sex and age distribution of the selected population matched the known epidemiology of CD. Following further validation, this case-finding algorithm may be useful for other claims database studies.

Study findings suggest that compared to CD-free controls, young CD patients have higher incidence rates of comorbidities such as cardiovascular, endocrine, musculoskeletal, and mental health conditions. Many of the comorbidity rates were two to five times higher than the matched controls. In addition, substantially higher health care resource use and costs were observed for CD patients compared to CD-free controls. Furthermore, even larger cost differences may have been observed if the sample had included healthier controls with no health care use as well. Earlier diagnosis, improved surgery success rates of cure, and better treatments may all help to reduce the chronic comorbidity and high health care costs associated with CD.

## Electronic supplementary material

Supplementary material 1 (DOCX 36 kb)
